# High depletion of breast cancer cells from the peripheral blood with the method of non-specific separation

**DOI:** 10.3332/ecancer.2020.1003

**Published:** 2020-01-21

**Authors:** Petra Payer, Michael Röder, Oumar Camara, Katharina Pachmann

**Affiliations:** 1 INNOVENT e. V., Prüssingstraße 27 B, D-07745 Jena, Germany; 2 Hufeland Klinikum GmbH, Rudolph-Weiss-Straße 1-5, D-99947 Bad Langensalza, Germany; 3 Transfusionsmedizinisches Zentrum Bayreuth, Kurpromenade 2, D-95448 Bayreuth, Germany

**Keywords:** breast cancer, circulating epithelial tumour cells (CETC), EpCAM-positive cells, magnetic nanoparticles, carboxymethyl dextrane (CMD) coating, non-specific cell separation, mild magnetic separation, external separation column

## Abstract

Circulating epithelial tumour cells (CETCs) play an important role in the formation of metastases in breast cancer patients. The depletion of such CETCs from peripheral blood of breast cancer patients using non-specific separation (without antibodies) of tumour cells from normal blood leucocytes might contribute to reduce the load of the patient’s blood with tumour cells and subsequently reduce the probability of metastasis formation. This method is based on cell type-specific interaction of living cells with Carboxymethyl Dextrane (CMD) coated magnetic nanoparticles. We have developed a mild flow separation method using CMD-coated magnetic nanoparticles (core size ca. 25 nm) along with a low-field gradient magnetic separator and an external separation column (blood bag). The ability of tumour cells to preferentially bind such particles and to separate tumour cells from the white blood cells from blood samples of 25 breast cancer patients (fresh and 24-hour stored blood samples) were tested. The circulating tumour cells were quantified before and after separation by maintrac analysis. We achieved a very high depletion rate of tumour cells to < 3% remaining in the investigated 24 hours stored blood samples and ≤14% in all fresh blood samples concurrent with maintaining 56% ± 4% of vital leukocytes in all fresh blood samples.

## Background

Breast cancer is the most frequent cancer for women in the developed world [1]. Most of the breast cancer patients, who die, do not die as a result of the primary tumour, however, due to later developing metastases. The dissemination of the cancer occurs by tumour cells, which can leave the primary tumour and migrate through the blood and lymphatic systems, as circulating epithelial cells [2]. It is hypothesised that these cells can be detected in the blood circulation already at an early stage of the disease.

The guidelines in breast cancer recommend chemo-, radio- and hormone therapy after surgery with the aim to eliminate occult residual disease such as circulating tumour cells and micro-metastases. Nevertheless, 25% of patients suffer a relapse [3]. This indicates that the adjuvant therapy methods in these cases are insufficient.

Circulating tumour cells may be of high relevance for diagnosis and monitoring of breast cancer. The detection of circulating tumour cells is currently based on different technologies [4–9]. There are at present two main methods: physical separation due to size, deformability or other physical properties of the cells, which can be followed by polymerase chain reaction (PCR) or other molecular diagnostic methods, and immunological assays which utilise monoclonal antibodies against surface antigens of the cells.

A therapeutic method based on these properties for depletion of tumour cells does not exist so far. Methods using ‘unspecific separation’ of tumour cells (without antibodies) from the white blood cells have shown promising results in this respect. The key point of this method is a selective interaction of tumour cells and leukocytes with Carboxymethyl Dextrane (CMD) coated magnetic nanoparticles. The CMD-shell and incubation conditions (time and milieu) play an important role for this cell type-specific interaction. Under defined conditions (incubation time, plasma concentration, etc.) tumour cells have shown a more intense interaction with the CMD-coated magnetic nanoparticles than white blood cells. With the use of magnetic forces it is possible to achieve a depletion of tumour cells from majority of healthy cells.

This non-specific separation method was developed by Clement *et al* [10]. This research group intensively studied the differential nanoparticle interaction [10–13] and utilised super-paramagnetic nanoparticles and magnetic separation in a high magnetic field gradient (Magnetic Activated Cell Sorting (MACS^R^), Miltenyi).

Our approach, in contrast, provides a mild separation of cells in order to maintain the viability of the normal remaining blood cells using a new flow separation method that makes use of magnetite nanoparticles with mean size of 25 nm along with a low field gradient and an external separation column (blood bag). Using the breast cancer cell line MCF-7 and leukocytes separately we optimised the conditions for incubation and separation and then artificial mixtures of MCF-7 cells with leucocytes were tested for separation efficiency [14]. Here, the applicability of our approach to remove tumour cells from the peripheral blood was tested in 25 blood samples from breast cancer patients.

## Methods and materials

### Magnetic nanoparticles

Magnetite-based nanoparticles were prepared by a wet chemical precipitation method using a partial oxidation of Fe(II) salt under a constant pH of 11 at 80 °C. The resulting nanoparticles were characterised by scanning electron microscopy (SEM), X-ray diffractogram and vibrating sample magnetometer (VSM). The particles consist of magnetite with a mean size of 25 nm. The saturation magnetisation of 73 emu/g is sufficiently high for our magnetic separation method.

The biocompatible coating with CMD—essential for this non-specific method —was carried out directly after preparing the nanoparticles. We applied pH 4.5 and 45 °C for this coating process. The use of ultrasound before and after coating was necessary to reduce aggregate formation. The produced magnetofluid was characterised with photon correlations spectroscopy (PCS). The hydrodynamic diameter was approximately 180 nm and the zeta potential was −56 mV. The Fe-content was determined by titration with KMnO_4_ and Na_2_S_2_O_3_, respectively. The Fe_3_O_4_-content was ca. 60 mg/mL (titration) and the CMD-content approxymately 4.4 mg/mmol Fe (photometric).

## Blood samples

Peripheral blood anticoagulated with ethylene diamine tetra acetate (EDTA) was drawn from 25 breast cancer patients with informed consent according to the Ethics Committee.

The circulating epithelial tumour cells (CETCs) were quantified before and after separation of analogous to the maintrac method [2], i.e., leukocytes containing tumour cells were prepared by erythrocyte lysis, labelled with fluorescence markers (Anti-Epithelial Cell Adhesion Molecule (EpCAM) and CD45) and analysed by a laser scanning cytometer (LSC CompuCyte, Cambridge MA, USA). We did not distinguish between the dead and living cells (no edition of propidium iodide).

**Figure 1.**
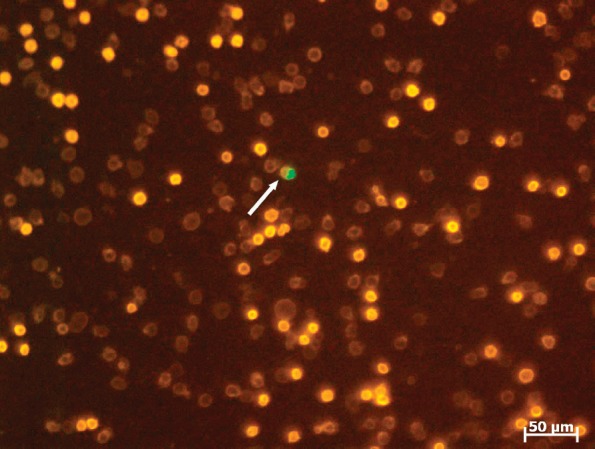
LSC fluorescence image of leucocytes (bright orange: lymphocytes, dim orange: granulocytes) including one tumour cell (arrow: cell with the green cap).

Specific details were as follows: Red blood cells from 1 mL of peripheral blood (anti-coagulated with EDTA) were lysed by adding 9 mL of erythrocytes lysis solution (Qiagen, Hilden, Germany) with an exposure time of 10 minutes at room temperature. The white cell pellet was then spun down at 300×g for 10 minutes and resuspended in 500 *μ*L PE buffer (phosphate-buffered saline (PBS) with 2 mmol/L EDTA). For fluorescence labelling, 5 *μ*L of fluorescein-isothiocyanate- (FITC-) conjugated mouse anti-EpCAM (Miltenyi, Bergisch Gladbach, Germany) and 1 *μ*L of phycoerythrin-(PE-) labelled anti-CD45 were added to 100 *μ*L of cell suspension, mixed well, then incubated for 15 minutes in the dark in the refrigerator and diluted with PE to a volume of 500 *μ*L. 50 *μ*L (100 *μ*L) of this cell suspension were pipetted onto the measuring area on a poly-L-lysine treated slide (Menzel Gläser, Braunschweig, Germany). The settled cells (after 10 minutes) were measured using a LSC (Compucyte Corporation, Cambridge, USA) and displayed in scattergrams and dot plots. Each EpCAM-positive event from the scattergram was visually analysed and confirmed as cells showing a green fluorescence cap in fluorescent light, shown in Figure 1.

With regard to future applications of this method as a therapeutic approach we tested ≥24 hours (day 1) stored blood samples as well as fresh blood samples (day 0). The stored blood samples were prepared —via erythrocyte lysis and fluorescence labelling—as mentioned above. The fresh blood samples could not be labelled in this way due to the masking of surface antigens. Therefore, the cells were treated with TWEEN 20 according to the method described by Hekimian [15] to demask EpCAM. 1 mL of fresh blood sample was incubated with 10 *μ*L TWEEN 20 (Sigma Aldrich) for 5 minutes at room temperature. Thereafter the erythrocyte lysis was carried out and followed by fluorescence labelling and quantification by LSC as described above.

## Magnetic separation method

### Incubation conditions

The magnetic separation method was performed using 2 mL of peripheral blood from breast cancer patients.

The lysing of the erythrocytes and spinning down the white blood cells was carried out as described above. The leucocyte pellet containing the tumour cells were resuspended in 2 mL PE buffer and 2.5% plasma was added. For magnetic labelling, the cell suspension was incubated with 16 *μ*L magnetofluid (as described above) for 10 minute at 37 °C.

### Magnetic separation conditions

**Figure 2.**
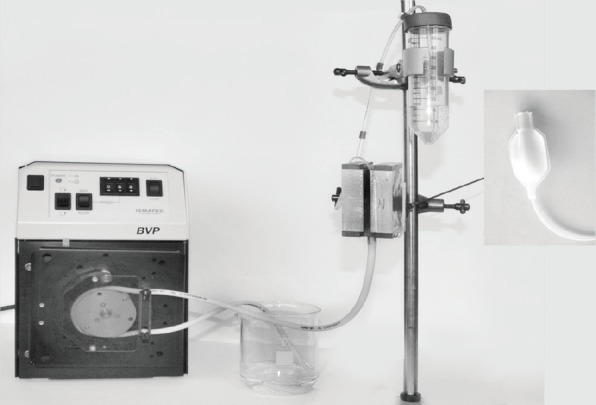
Experimental setup of the magnetic separation device (with cell pump, magnetic arrangement, blood bag and falcon tube for negative fraction).

The magnetic separator consists of a magnetic arrangement with a blood bag inside (separation column) (Figure 2). The labelled cell suspension was gently pumped in PE buffer medium through the blood bag (3 cm^3^) using a modified cell pump (ismatec, Wertheim, Germany). The cell suspension, which passed the separator, was designated as negative fraction (effluent). The cell suspension, retained in the blood bag, was designated as positive fraction and was eluded from the blood bag after removing it from magnet and by massaging it. Each fraction was collected in 50-mL falcon tubes with the addition of 10-mL Dulbecco’s Modified Eagle Medium (DMEM) plus 10% fetal calf serum.

## Analysis of the negative and the positive fraction

The cell fractions were spun down and the cell pellets were resuspended in 1-mL PE. The cell counts of leukocytes were analysed by Cell Scepter (Merck Millipore). The CETCs in the negative fractions were quantified in analogy to the Maintrac method by labelling with fluorescence markers and analysed by LSC as mentioned above. The negative fractions of fresh blood samples had to be treated with tween (analogous to the whole fresh blood) before labelling with fluorescence markers. The reaction was stopped by adding a PE-buffer.

The vitality of leukocytes was controlled with live/dead staining (fluorescein diacetate (FDA)/GelRED). Fluorescein diacetate freely enters the intact cells and is converted by the enzymes to fluorescein intracellularly. The cytoplasm of the vital cells is stained in green. GelRed penetrates the membrane of the dying/dead cells and reacts with nucleic acid of the cell nucleus and fluoresces red. The cells were incubated with the combined staining solution of FDA/GelRED (15 *μ*g/mL FDA and 1:10,000 dilution of GelRED stock solution) at room temperature for 3 minutes and examined under fluorescent light microscopy.

## Results

### Initial CETCs

Patients were numbered sequentially according to inclusion into the study (Tables 3 and 4). The enumeration of the CETCs pre-separation was correlated to the patient data (Table 1). All patients were without distant metastases.

Low, middle and high concentration of CETCs could be distinguished. Low numbers of epithelial cells (<2000 cells/mL) were observed in patients without lymph nodes involvement, including one patient (sample 15) with nodes had been removed at primary surgery and one patient with estrogen positive tumour after neoadjuvant chemotherapy (sample 25).

Patients with medium cell numbers were mainly patients with invasive ductal carcinomas apart from one with DCIS.

All triple negative (TN) invasive-ductal carcinomas (sample 3 equal 24, 19 and 20) were in the group with high numbers of circulating epithelial cells in contrast to the TN medullary carcinoma (sample 22) which had a low number of circulating epithelial cells. A neoadjuvant therapy obviously leads to a reduction of the initially high numbers in sample 3 (before neoadjuvant therapy) as compared to sample 24 (after neoadjuvant therapy).

Surprisingly, a high number of CETCs was observed in two patients with DCIS (samples 7 and 13).

It has been shown previously (Hekimian) that the EpCAM molecule may be masked in freshly drawn blood samples but can be made accessible to the respective antibody after a short treatment of the white blood cells with tween. Ten samples (Table 2) were analysed for the number of epithelial cells after treatment of the freshly drawn samples with tween. Eight samples could be analysed but in two TN tumour tween treatment of the freshly drawn blood samples (19 and 20) resulted in unspecific dying. This made the evaluation of the number of EpCAM positive cells impossible.

## Fresh blood compared to 24 hours stored blood samples

After 24 hours the TN fresh blood samples 19 and 20 were quantifiable. These patients, both, had high numbers of CETCs in the 24 hours stored samples without tween treatment indicating that these patients’ cells were especially vulnerable. Sample 20 was tested twice (day after 1st surgery and 2 weeks post 2nd surgery) with the same result.

We found a lower numbers of tumour cells with the storage (24 hours) of the blood samples in the samples 10, 18 and 22. This might, in part be due to short-lived wound healing cells (also EpCAM-positive cells) in the fresh blood samples and these will decrease during storage of the blood samples. In the samples 12, 13 and 21 the results showed an increase of CETCs after storage of the samples. TWEEN-treatment may have been insufficient here and so parts of surface antigens still remained masked.

One patient presenting for breast reconstruction after adjuvant radio-/chemotherapy 3 years ago (sample 17) was only tested in fresh blood sample.

## Magnetic separation of ≥24 hours stored blood samples

Magnetic separation was performed in 18 blood samples (16 post-surgery and 2 pre-surgery) from breast cancer patients in different stages of the disease. The blood samples had been kept at room temperature for 24 hours for observation so that EpCAM on the cell surface is partly masked and becomes accessible to the antibody only after at least 24 hours of storage. The timing after surgery was chosen, because surgery is the first inevitable intervention after the diagnosis of a malignant tumour. The interval between surgery and blood collection differed between right before surgery and 5 weeks after surgery (Table 3).

The magnetic separation method of this blood samples was carried out in the following steps: erythrocyte lysis, incubation of the cell suspension with magnetic fluid, tempering at 37 °C during incubation and followed by the magnetic separation as already described.

The separated cells were collected in the negative and positive fraction. Immediately after the end of the separation, the number of leukocytes in both fractions and the number of CETCs in the negative fraction were determined.

**Figure 3.**
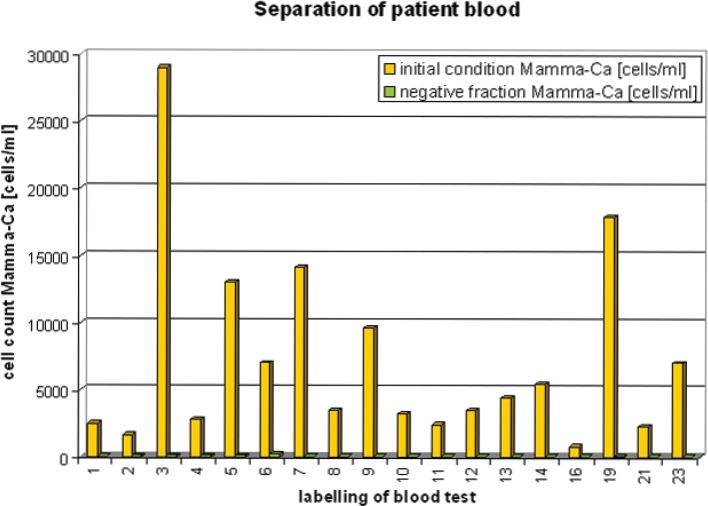
Stored patient blood (≥24 hours) before and after magnetic separation.

As compared to the pre-separation CETC counts—less than <3% CETCs remained in the negative fraction in all blood samples (Figure 3), with the exception of sample 2 <6% CETCs (no more accurate determination was made). Significant numbers of CETCs were only detected in the negative fractions of samples 5, 6 and 19. All the other results were from <25 to <100 cells/mL (Table 3). Thus, a very high depletion of tumour cells was achieved independent from the initial number of circulating epithelial cells in the blood samples.

## Magnetic separation of fresh blood samples

For future development of our method in the direction of therapeutic method, it was necessary to test fresh blood samples, 1–8 hours after the drawing of blood. From ten of the patients fresh blood samples were analysed. The magnetic separation was carried out with eight blood samples. The intervals between surgery and blood collection and the initial number of CETCs cells of these samples are listed in Table 4. It has already been mentioned, that labelling of fresh blood samples for EpCAM-expression may not represent the true numbers present in a sample due to the masking of surface antigens. Therefore, TWEEN-treatment [15] was applied to freshly drawn samples in order to demask EpCAM. The pre-separation numbers of CETCs varied widely.

Magnetic separation of the fresh blood samples was performed in the identical way as for the 24 hours stored samples, however, without the addition of TWEEN. This approach was only necessary for the detection of EpCAM positivity in the pretreatment samples as well as the determination of CETCs in the negative fraction.

**Figure 4.**
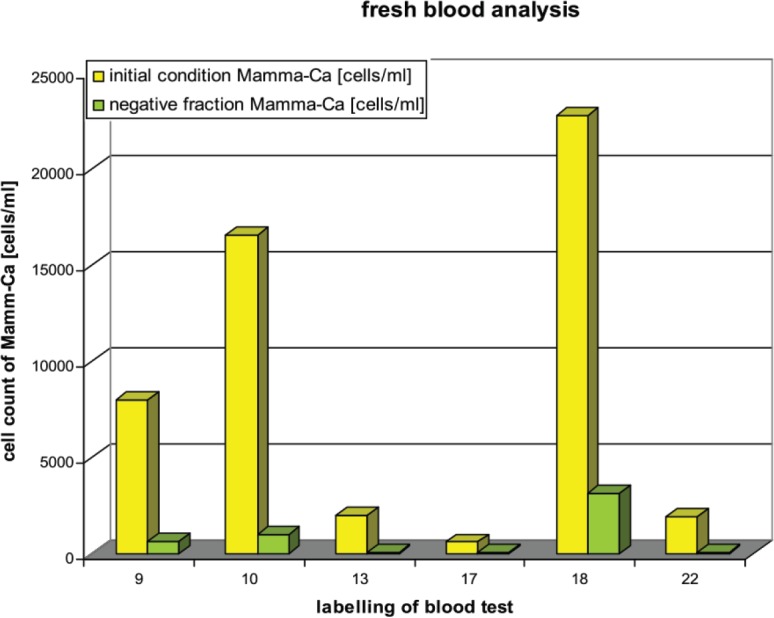
Fresh patient blood before and after using magnetic separation method.

As mentioned above in two of the freshly drawn blood samples 19 and 20 the number of CETCs could not be determined. Therefore, they are not shown in Figure 4.

Magnetic separation in the fresh blood samples resulted in ≤14% of tumour cells retained in the negative fraction.

## Influence of the separation procedure on leukocytes

**Figure 5.**
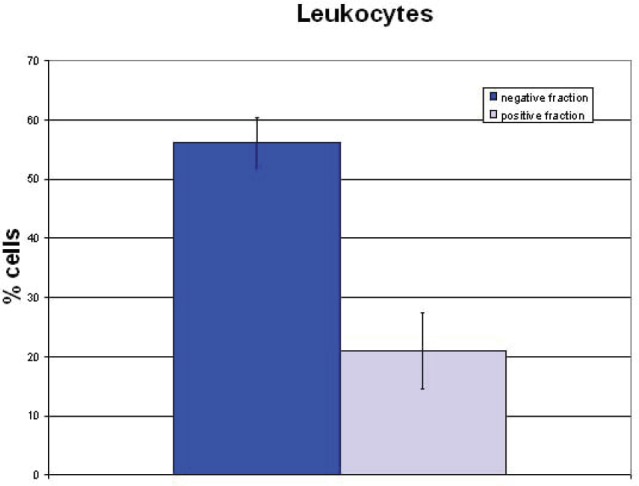
Cell yield of leukocytes in fresh blood samples.

**Figure 6.**
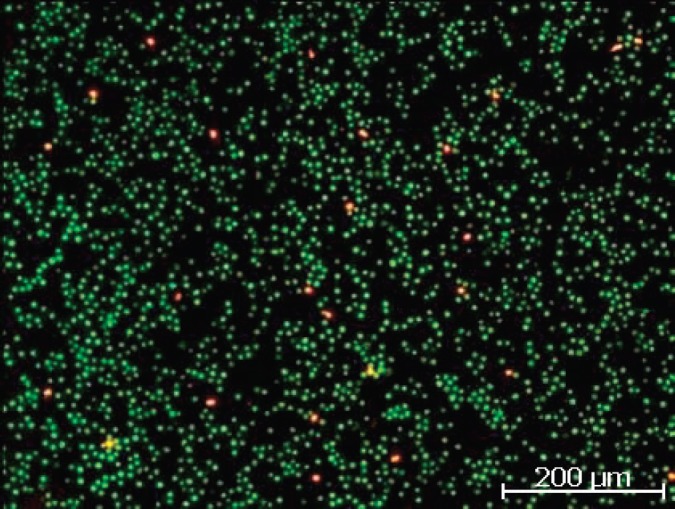
Vital leukocytes (green) after separation.

The behaviour of the normal blood cells before and after separation was also analysed with respect to quantification and vitality. The negative fraction of the **≥**24 hours stored blood samples contained 10% fewer leukocytes in the negative fraction and a corresponding 10% higher loss. In the negative fraction of the fresh blood samples 56% ± 4% leukocytes were retained, whereas in the positive fraction 21% ± 7% leukocytes were retained (Figure 5). Cell loss from the separation process amounted to 23%. This result could be expected because 70% of the white blood cells are granulocytes with a short lifetime. Live/dead staining (FDA/GelRED) to test the vitality of leukocytes in the negative fraction showed a high vitality of leukocytes and only a small amount of dying/dead cells (Figure 6).

Annexin/propidium iodide (PI) apoptosis necrosis detection kit (here not shown) showed that the lymphocytes retained a high vitality resulting in a selective enrichment of these cells in the negative fraction with only a small fraction of apoptotic/necrotic lymphocytic cells.

## Discussion

EpCAM is expressed on 90% of epithelial cancer cells on the basolateral surface of the cells [15] and can be detected by anti-EpCAM antibodies. Using anti-EpCAM-FITC in analogy to the standardised maintrac method [2] we have determined CETCs in the blood of breast cancer patients.

Maintrac is a fast and sensitive quantitative method for the detection and characterisation of tumour suspected cells in the blood. Different studies have shown that the behaviour of epithelial tumour cells in the breast cancer blood can serve to predict the subsequent course of the disease [16–21]. A decrease in the number of cells during systemic therapy highly significantly correlates with the response to the therapy. On the other hand an increase in the number of circulating tumour cells indicates an increasing risk of formation of metastases and relapse.

Therefore, depletion of EpCAM-positive cells from the peripheral blood of breast cancer patients with a non-specific separation method might be able to reduce the probability of metastasis formation. It needs to be emphasised that the magnetic labelling of the cells took place without antibodies. Antibodies were only used for the quantitative analysis of the cells. We used CMD-coated magnetite-based nanoparticles (mean size Ø 25 nm) for the magnetic labelling which allowed a low-field gradient separation. It is a mild magnetic flow separation method with a blood bag—separation column—inside the permanent magnetic arrangement.

## CETCs

Using the maintrac method in most of the 25 breast cancer patients blood samples were drawn around surgery (exception sample 17). In 24 samples CETCs were detected. Only in one sample (sample 25) we did not find any CETCs with our method (<100 cells/mL). In this case, the sampling occurred immediately after the end of neoadjuvant therapy.

It has been shown, that the number of CETCs changes with the interval between surgery and sampling. There is an increase right after surgery and a re-decrease until day 7 after surgery [22]. We could compare repeated sampling in the cases of sample 7, 19 and 20. With the samples 7 and 19 the number of CETCs was in the same range at the day of surgery or following day and 1 week post-surgery. But in the case of sample 20, we found a 4-fold higher number of CETCs after the second surgery as compared with that after the first.

Testing freshly drawn blood showed that the detection of CETCs is only possible with previous TWEEN-treatment before as demonstrated in the samples 1, 2, 10, 13, 17 and 18 where we could not find any CETCs without TWEEN-treatment (not shown).

Repeated testing in the same samples as freshly drawn blood and as 24 hours stored samples (samples 12, 13, 18, 19, 20, 21 and 22) showed a decrease of tumour cells with the storage in samples 18 and 22.

**Figure 7.**
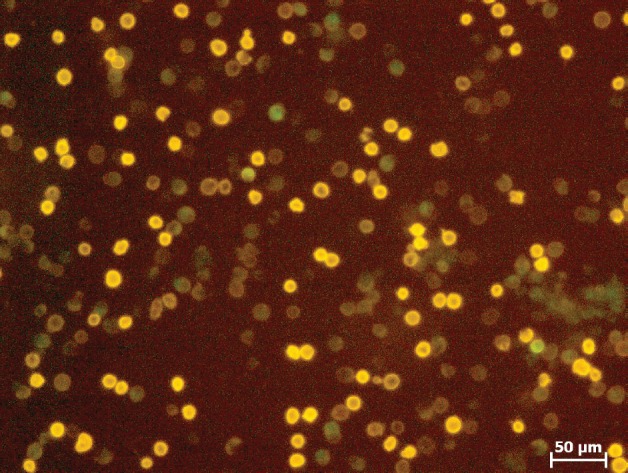
Sample 19 fresh blood.

**Figure 8.**
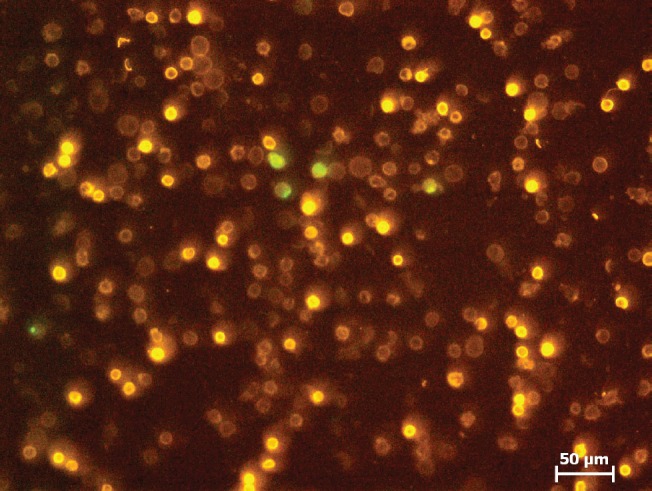
Sample 19 ≥24 hours stored blood.

It is noteworthy that we detected a lot of small fully coloured EpCAM-positive cells in the fresh blood sample which were no longer detectable after the 24 hours storage period. They may represent short-lived wound healing cells which are eliminated during the storage time. In the fresh blood samples 19 (Figure 7) and 20 (two tests—following day 1.surgery + 2 weeks post 2.surgery) we found unspecific dying with EpCAM precluding correct enumeration. Both samples were from triple receptor negative (TN) breast cancer patients. After storage of blood for 24 hours (e.g. Figure 8) we were able to quantify the CETCs. This interesting aspect must be verified.

## Magnetic separation method

We achieved a high depletion of tumour cells in all the tested blood samples. This result was independent from the initial number of CETCs and tumour staging and grading. Thus, the absolute number of CETCs seems not to be critical for our separation method.

There were, however, differences in the separation of fresh blood and **≥**24 hours (1 day) stored blood samples. In the 1 day stored blood samples we were able to remove >97% of CETCs by using the non-specific separation method. Whereas the depletion of tumour cells in the fresh blood samples was **≥**86%. The differences in the separation of fresh blood and ≥1 day stored blood might be explained assuming that not all EpCAM-positive cells necessarily are malignant cells. Benign EpCAM-positive cells may be released into the blood during, e.g., wound healing due to surgery. These cells may be short-lived cells and disappear within 1 day. The endocytosis of magnetic nanoparticles by such cells may be slower than that of the malign cells and as a consequence they cannot be fully removed with this method. In the future, gene analysis of individual cells might be helpful to clarify the nature of such EpCAM-positive cells and future tests with fresh blood, not in relation to surgery, will be needed. Under these conditions, the situation is expected to be similar (number of CETCs) in fresh blood and 1 day stored blood.

In all our tests, we could show that the magnetic separation method developed by us may become a useful tool for the successful depletion of CETCs from the blood of breast cancer patients. Until now, the tests were performed in a low scale. We now are in the process of scaling up the method with a new blood bag (100 cm^3^) and a new magnetic separator using MCF-7 and the cell line SD-1 in a model system to further develop the method using a blood volume of 60 mL and optimising the separating conditions and continued our tests with cell mixtures of leukocytes (buffy coats) and MCF-7 are ongoing.

The removal of such cells with this unspecific separation method may provide a possibility to reduce the formation of metastases and offer an opportunity to reduce the number of relapses. This method exerts little mechanical stress (cell pump) to the healthy cells. The potential return of the healthy cells (in particular lymphocytes) may be very important for maintaining the immunological competence of the patients. This is also very important for the further health of the patients. The preservation of the immune system plays an exceedingly important role in the control of malignant tumours. This has been shown by the recent therapeutic success of check-point inhibitors.

## Conclusion

The developed method of mild magnetic separation is suitable for the successful depletion of CETCs from the blood of breast cancer patients (ex vivo) while maintaining a large proportion of vital healthy cells. This method has a high potential for future therapeutic applications in the adjuvant therapy.

We have scaled up this method and intend to test it in animal studies.

## Conflict of interest

The authors have no conflicts of interest.

## Funding

This work was supported by the BMWi-Programme INNO-KOM-OST MF 110033.

## Figures and Tables

**Table 1.** Preseparation of epithelial cell numbers of patients and correlation to tumour stage (≥24 hours blood).

**Low numbers of epithelial cell numbers (<2000 cells/mL)**			
**Blood sample**	**Histology**	**Tumour formula**	**Epithelial cell number/mL blood**
2	DCIS	N0, G3, ER+, PR+, HER2 n.e.i.	1,600
15	Invasive-lobular	rpT3, G3, N removed (primary surgery), ER+, PR+, HER2−	1,000
16	Invasive-ductal	T1b, N0, G1, ER+, PR+, HER2−	1,100
22	Medullary	T2, N0, G3, triple negative tumour, medullary	800
25	Invasive-ductal	yT2, N0, G2, ER+, PR−, HER2+	<100
**Medium content of epithelial cell numbers (2000–4000 cells/mL)**			
**Blood sample**	**Histology**	**Tumour formula**	**Epithelial cell number/mL blood**
1	Invasive-ductal	T1c, N2a, G3, ER+, PR+, HER2+	2,500
4	Invasive-ductal	T1c, N0, G2, ER+, PR+, HER2−	2,600
8	Invasive-ductal	T1c, N1a, G2, ER+, PR+, HER2−	3,500
10	DCIS	DCIS,N0, G2, ER+, PR−, HER2 n.e.i.	3,200
11	Invasive-ductal	T1c, pN1a, G1, ER+, PR+, HER2−	2,400
12	Invasive-ductal	rpT1c, pN0, G3, ER+, PR-, HER2+	3500
**Blood sample**	**Histology**	**Tumour formula**	**Epithelial cell number/mL blood**
21	Invasive-ductal	T3, N1a, G3, ER+, PR+, HER2+	2,800
26	Invasive-ductal	T2, N1a, G2, ER+, PR+, HER2−	3,300
**High content of epithelial cell numbers (>4000 cells/mL)**			
**Blood sample**	**Histology**	**Tumour formula**	**Epithelial cell number/mL blood**
5	Invasive-ductal	T2,N1a, G2, ER+, PR+, HER2−	13,000
6	Invasive-ductal	T1c/ T2, N0/ N1a, G2, ER+, PR+, HER2−**	7,000
7*	DCIS	DCIS, G2, N and hormonal status n.e.i.	15,800/12,400
9	Invasive-ductal	T1c, N1a, G1, ER+, PR+, HER2-	8,000
13	DCIS	DCIS, G3, N and hormonal status n.e.i.	4,400
14	Invasive-ductal	T2/ T1c, N0/ N2a, G2, ER+, PR+, HER2−**	5,900
18	Invasive-lobular	T1c, N1mi, G2, inv. lob. T., ER+, PR+, HER2−	11,800
19*	Invasive-ductal	T1c, N0, G3, triple negative tumour	15,000/18,000
20	Invasive-ductal	T2, N3a, G3/ pT3, N1a, G3, triple negative tumour**	8400
23	Invasive-ductal	T2, N0, G2, ER+, PR+, HER2−	6,000
3	Invasive-ductal	cT2, N1a, G3, triple negative tumour	29,000
24 same patient as 3	Invasive-ductal	yT1b, G3, triple negative tumour	4,200

* tested twice

** bilateral

n. e. i. not elsewhere identified

**Table 2.** Initial epithelial cell numbers of patients and their tumour data (fresh blood).

Blood sample	Histology	Tumour formula	Epithelial cell number/mL blood
9	Invasive-ductal	T1c, N1a, G1, ER+, PR+, HER2−	9,600
10	DCIS	DCIS,N0, G2, ER+, PR−, HER2 n.e.i.	16,600
12	Invasive-ductal	rpT1c, N0, G3, ER+, PR−, HER2+	300
13	DCIS	DCIS, G3, N and hormonal status n.e.i.	2,000
17		Only breast reconstruction	600
18	Invasive-lobular	T1c, N1mi, G2, inv. lob. T., ER+, PR+, HER2−	22,800
19	Invasive-ductal	T1c, N0, G3, triple negative tumour	Non-quantifiable
20*	Invasive-ductal	T2, N3a, G3,/ pT3, N1a, G3, triple negative tumour**	Non-quantifiable/non-quantifiable
21	Invasive-ductal	T3, N1a, G3, ER+, PR+, HER2+	900
22	Invasive-ductal	T2, N0, G3, triple negative tumour, medullary	1,900

* tested twice

** bilateral

**Table 3.** Overview of the ≥24 hours stored blood samples.

Patient samples (1 day)	Blood collection	Initial condition EpCAM + cells/mL blood	Negative fraction EpCAM + cells /mL blood
1	5 weeks post-surgery	2.500	<25
2	5 weeks post- surgery	1,600	<100
3	Before neoadjuvant treatment	2,9000	<100
4	Day after surgery	2,600	<30
5	2 days post-surgery	13,000	100
6	Day of surgery	7,000	50
7	Day of surgery*/1 week post- surgery	15,800/12,400	<25
8	2 weeks post-surgery	3,500	<25
9	Day of surgery	8,000	<25
10	1 week post 1st surgery	3,200	<25
11	Pre surgery (day of surgery)	2,400	<25
12	2 days post-surgery	3,500	<25
13	2 weeks post-surgery	4,400	<25
14	Pre surgery (day of surgery)	5,900	<25
15	Day after surgery*	1,000	-
16	Day after surgery	1,100	<25
18	Day after surgery*	11,800	-
19	Day after surgery*/ 1 week post-surgery	15,000/ 18,000	50
20	Day after 1st surgery*/2 weeks post 2nd surgery*	1,800/8,400	-
21	Day after surgery	2,300	<25
22	Day of surgery*	800	-
23	1 week post-surgery	6,000	<25
24	1 week post-surgery*	4,200	-
25	Day after surgery*	<100	-
26	Day after surgery*	3,300	-

* only detection of CETCs, no separation

**Table 4.** Overview of the fresh blood samples.

Patient samples (fresh blood)	Blood collection	Initial condition EpCAM + cells /mL blood	Negative fraction EpCAM + cells /mL blood
9	Day after surgery	9,600	650
10	Day of 2nd surgery	16,600	1,000
12*	2 days post-surgery	300	-
13	2 weeks post-surgery	2,000	50
17	Day after surgery for breast reconstruction	600	50
18	Day after surgery	22,800	3,100
19	1 week post-surgery	non-quantifiable	3,450
20	Day after surgery*/2 weeks post 2. surgery	non-quantifiable/ non-quantifiable	3,300
21*	Day after surgery	900	-
22	Day of surgery	1,900	50

* only detection of CETC, no separation
